# Treatment of Chorea by the Use of Cannabis Indica

**Published:** 1845-09

**Authors:** 


*Treatment of Chorea by the Use of Cannabis Indica.*—This medicine has been administered with great success by Dr. Corrigan, in some cases of chorea, at the Richmond Hospital, the details of which are given in the Hospital Gazette. The subject of the first case was a girl, aged 10, in whom the muscles of the upper and lower half of the body, together with those of the tongue and face, were engaged. It had been of five weeks' standing on her admission, at which time she was ordered gtt. v. tr. can. Ind., ter in die; considerable amendment had taken place in eleven days, and the dose was then increased to gtt. xv., ter in die. This quantity she continued to take for a little more than five weeks, when she was discharged cured.

In a second case, that of a girl, aged 14, in whom the affection was confined to the left side of the body, it had commenced a month before admission, and after an oil draught, with turpentine, had been given, she was put upon doses of eight drops of the tincture three times a-day. The quantity was gradually increased to gtt. xxv. ter in die, the patient complaining of headache and lightness of the head after each dose. At a period of six weeks from the time of her admission, she was discharged cured.

A third case, occurring in a girl, aged 16, had been of 10 years' standing at the time of her admission. She had been subjected to treatment at two hospitals previously, with slight amendment on each occasion. The affection here was more marked in the muscles of the arm than in those of the lower extremities, the patient being able to walk steadily. The affection in this case was ascribed to fright, but in neither of the foregoing ones could the patients attribute it to any particular cause. This girl took ten drops of the tincture, three times daily, and was discharged at the end of a month.

Dr. Corrigan also records the case of a lady, who had long suffered from severe neuralgia of the face, neck, and head, arising from cold. On several occasions she had found relief from the use of tonics, and the application of liniments.

From a very severe attack following influenza, Dr. Corrigan ordered gtt. xx. tr. can. ind. ter die. The first dose, however, produced the following effects :—inability to swallow in half an hour after; could not keep her eyes open, though perfect consciousness of all that was passing around her remained ; she then fell asleep for some hours, and on awaking felt a slight twitch in the left cheek. This occurred on the 19th of April last, since when she has been quite well.

In his observations on these cases, Dr. Corrigan goes into the question of the cause of chorea, and remarks, that from its greater frequency in females, it might be supposed to depend on the condition of the uterus, but that the disease is most often met with between the age of 7 and 14. He objects to its connection with cerebral derangement, because, he says, the subjects of the affection are remarkably intelligent, and the functions of the brain are undisturbed during the attack. It cannot, he considers, be traced to disorder of the digestive functions, as these are commonly in good condition, and accompanied sometimes with ravenous appetite—a feature that was present in the third case of chorea, alluded to above.

Viewing the negative and positive symptoms of the disease, then, he says, it may be looked on as a mere functional derangement of the motor nerves, either of the brain or spinal column, more frequently seen in females, because of the more excitable condition of the nervous system in them. Dr. Corrigan contrasts the effects of the Indian hemp with that of aconite, the action of the former being primarily on the motor nerves, its influence, he inclines to think, being transmitted along these to the sensorium and nerves of sensation, as in the case of tic doloureux, just alluded to in the lady, while the action of aconite is exactly the converse.

Speaking of the peculiar advantages of Indian hemp as a sedative, he assures us that even in over-doses it does not produce the dry tongue or derangement of the digestive organs, such as is seen to follow the use of opium ; its effects on different individuals, however, he remarks are very variable ; in the case of the lady for instance, twenty drops of the tincture caused temporary loss of power in almost all the muscles, followed by sleep, while a similar dose has been taken by other patients three times daily for weeks with impunity and advantage.

Dr. Corrigan thinks that in all cases ten drops may be given three times a day, increased after the third day to twenty or even thirty drops, but more than this he has never ventured to give ; by mistake fifty drops were given instead of five to patients laboring under rheumatism and arthritic pains, for whom he had ordered the latter dose. In these cases the overdose produced severe headache, and what some of the patients described as white sight, but gave no relief to the pains.

Dr. Corrigan also alludes to the advantages derivable from the moderate use of electro-magnetism, when in the progress of treatment, the patient, apparently on the point of recovery, suddenly ceases to advance; this he has not known to occur in the treatment by Indian hemp, but frequently under the use of other remedies.—*London Med. Times.*

